# Primary Prevention of Heart Failure

**DOI:** 10.5402/2012/982417

**Published:** 2012-08-16

**Authors:** Javed Butler

**Affiliations:** Emory University, Atlanta, GA 30322, USA

## Abstract

Most heart failure research and quality improvement efforts are targeted at treatment and secondary prevention of patients with manifest heart failure. This is distinct from coronary disease where primary prevention has been a focus for over three decades. Given the current importance and the projected worsening of heart failure epidemiology, a more focused effort on prevention is urgently needed.

## 1. Epidemiology

It is estimated that over 5.5 million subjects in the United States have heart failure and more than 650,000 are diagnosed for the first time each year [1]. Patients with impaired versus preserved left ventricular systolic function related heart failure each comprises about half of the overall burden of heart failure in the community [2, 3]. The proportion of heart failure with preserved ejection fraction increases with age [2]. Heart failure is the primary reason for 12–15 million office visits and 6.5 million hospital days annually. Recurrent hospitalization is a major issue with the annual number of hospitalizations now exceeding over 1 million for heart failure. These patients are particularly prone to rehospitalizations with readmission rates near 50% within six months of discharge. It has been estimated that the total direct and indirect cost of heart failure in the United States exceeds $30 billion [1]. The outcomes of these patients continue to remain suboptimal with only approximately 50% of the individuals surviving past five years after diagnosis [4]. Quality of life remains poor. Some improvement have been shown in individuals with systolic dysfunction, with no major advances in therapy for either patients with heart failure and preserved ejection fraction or those who are hospitalized for heart failure.

Heart failure prevalence is rising and this trend will worsen. This is attributed to the increasing elderly population and the increasing prevalence of cardiovascular risk factors like diabetes and obesity. The aging of the 78 million baby boomers will result in 1 in 5 Americans to be over the age of 65 years by 2050. Heart failure incidence and prevalence are the highest amongst the elderly, and 80% of patients hospitalized with heart failure are over 65 years old. Thus the increasing age of the population is expected to significantly worsen the current heart failure epidemic. 

## 2. Risk Factors 

Risk factors for heart failure range from lifestyle factors to comorbidities, medications, laboratory, and imaging characteristics to novel biomarkers and genomic markers [5]. Heart failure risk increases with age and male gender is associated with a higher risk [6]. Lower physical activity, coffee consumption, increased salt intake, and lower socioeconomic status have been associated with increased risk [6]. Hypertension, diabetes, obesity, and coronary disease all increase risk. More than half of the patients admitted for heart failure regardless of ejection fraction have coronary artery disease [7]. Hypertension and coronary artery disease are the most common and strongest risk factors conferring a 2- to 3-fold increased risk [8]. Valvular heart disease increases risk through hemodynamic alterations. Obesity, through multiple mechanisms, predisposes to heart failure [9]. Excessive alcohol intake increases blood pressure and is a direct myocardial toxin [10]; however, light-to-moderate consumption has been inversely associated with risk, especially in men [11, 12]. Smoking promotes several cardiovascular risk factors associated with heart failure [4, 6]. Dyslipidemia and renal dysfunction predisposes to heart failure [13–16]. Other comorbidities that increase risk include anemia, sleep disordered breathing, increased heart rate, pulmonary dysfunction, and microalbuminuria. Levels of homocysteine and natriuretic peptide are all associated with an increased risk. Serum resistin [17], lipoprotein associated phospholipase A_2_ [18], and myeloperoxidase levels [19] have been associated with increased risk also.

Several chemotherapeutic agents, for example, doxorubicin, cyclophosphamide, trastuzumab, and 5-fluorouracil are associated with heart failure. Cyclooxygenase-2 inhibitors may increase risk of myocardial infarction. Thiazolidinediones have been associated with edema and precipitation of heart failure [20]. Several cardiac anatomic and physiological measures are associated with a higher risk including chamber dilatation with an increase in end-diastolic or end-systolic dimensions, increased left ventricular mass, left ventricular diastolic filling impairment, left atrial enlargement, and asymptomatic systolic dysfunction. There is growing interest in the genomic predictors of heart failure [5]. Genetic polymorphisms in sympathetic receptors, for example, *α*_2C_ adrenergic receptors (*α*_2C_ del322–325) or *β*_1_ adrenergic receptors (*β*_1 _Arg389) are associated with heart failure. Homozygous blacks for *α*_2C_ del322-325 have a 5-fold higher risk. If this polymorphism is associated with homozygosity for *β*_1 _Arg389, the risk increases by 10-fold.

 Population attributable riskrepresents the proportional reduction in disease risk that would be achieved by eliminating the risk factor from the population, assuming a causal relationship. In a recent report [21], coronary heart disease and uncontrolled blood pressure were the leading causes of heart failure in whites and blacks and in men and women. A substantial proportion of heart failure, however, was also attributed to metabolic and cardio-renal factors, including glucose and renal abnormalities. Severalprevious investigations have reportedsubstantial sex- and race-related differences in population attributable risks and disease development for heart failure [4, 22].Understanding and quantifying these differences is important for planning appropriate preventive interventions.The higher incidence of heart failure in black compared to white participants in the Health ABC study was simultaneously accompanied by a higher prevalence of risk factors in black participants. These data provide valuable information into race-based differences and helps prioritize interventions, set targets, and assess the feasibilityof novel therapies.

## 3. Risk Prediction

Although many heart failure risk factors have been described, determining their role in predicting a future event is still challenging. Despite strong etiologic association with a disease, a risk factor may be limited in its prognostic role [23]. Although individual risk factors for heart failure, for example, hypertension, are well described, how to quantify individual risk in patients with various combinations of risk factors is not clearly described. Multiple risk factor prediction schemes, for example, the Framingham Risk Score, have been developed for coronary events [24]. However, the heart failure syndrome represents a spectrum ranging from ischemic to nonischemic etiologies and normal to depressed ejection fraction. Elderly subjects may develop heart failure due to age-related cardiovascular changes in the absence of traditional risk factors. High-risk subjects therefore may not be detected using coronary risk schemes. For example, in the cardiovascular health study, 66% incident heart failure developed in subjects without baseline coronary heart disease and more than half never had a preceding coronary event prior to developing heart failure [25]. 

Most studies on heart failure risk factors have studied individual risk factors.  Table 1 summarizes the studies that have assessed independent risk factors comparing multiple risk factors [6, 25–28, 31–34], and only two of them developed a prediction model for incident heart failure [28, 34]. The Framingham Heart Failure Risk Score assessed the probability of developing heart failure over a 38-year followup among 6354 person examinations in men and 8913 in women. Although regression coefficients differed among men and women somewhat, overall predictors were similar. Interestingly, systolic blood pressure was only predictive in men and diabetes mellitus in women. The investigators also developed a 4-year event score with an event rate averaging 3.97 per 100-person year in men and 2.63 in women with a 37% increment per decade of age. The chest X-ray and pulmonary function test requirement makes it more difficult to implement it in large population settings for screening. The model was also drawn primarily on Caucasian population. The model was derived on a restricted group of subjects with a known history of either hypertension or coronary heart disease or valvular heart disease; such characteristics represented less than 50% of the population in other cohorts, for example, the Health Aging, and Body Composition (Health ABC) study [34], limiting the model's generalizability. 

Recently, Health ABC Heart Failure Risk Model was developed using the data from 2935 individuals participating in the health ABC study. Independent predictors of heart failure included age, history of coronary heart disease and smoking, systolic blood pressure and heart rate, serum glucose, creatinine, and albumin levels, and electrocardiographic left ventricular hypertrophy; the model has good discrimination and calibration. A simple point score was created to predict incident heart failure risk into 4 risk groups corresponding to <5%, 5% to 10%, 10% to 20%, and >20% 5-year risk was developed. The investigators externally validated the model in the cardiovascular health study; the model retained adequate predictive capabilities [35]. The model predicted risk equally well in both men and women, and in white and black races.

## 4. Challenges in Risk Prediction 

Several unique issues make heart failure risk assessment challenging. First, heart failure is a clinical diagnosis and this leads to diversity in opinions and diagnostic uncertainty in a proportion of cases. The most common clinical criteria used to diagnose heart failure is the Framingham criteria that requires the presence of at least two major, or one major and two minor criteria [36]. Major criteria include paroxysmal nocturnal dyspnea, neck vein distension, rales, radiographic cardiomegaly, acute pulmonary edema, S3 gallop, increased central venous pressure >16 cm H_2_O, circulation time ≥25 seconds, hepato-jugular reflux, or pulmonary edema or visceral congestion or cardiomegaly at autopsy. Minor criteria included bilateral ankle edema, nocturnal cough, dyspnea on ordinary exertion, hepatomegaly, pleural effusion, reduced vital capacity by one third from maximum, and heart rate ≥120 beats/minute. Investigators from the Cardiovascular health study developed alternative criteria that included medication use and imaging modalities [37]. When both sets of criteria were compared, only half the patients were adjudicated as having heart failure by both criteria, whereas the other half was labeled with either one or the other, but not both criteria [38]. Similar discordance has been shown between administrative discharge diagnoses versus based on detailed chart review [39]. 

Part of this discordance is related to diagnosing heart failure with preserved ejection fraction. Many of the symptoms (e.g., shortness of breath) and signs (e.g., edema) are nonspecific and can be seen in other conditions for example obesity or chronic lung disease. The European Society of Cardiology developed a consensus statement for diagnosing this condition using biomarker and imaging-based detailed protocols [40]. Similarly, another set of detailed clinical criteria to diagnose incident heart failure in clinical trials was recently published [41]. These criteria are of limited usefulness from population perspective. 

## 5. Risk Modulation

Currently, incident heart failure risk modulation is primarily targeted towards risk factor management individually. For coronary artery disease, patient treatment plan is targeted based on the individualized risk profile related to the various combinations of risk factors, for example, blood pressure goals or low-density lipoprotein level. On the contrary, no such paradigm for heart failure risk modulation currently exists. Many heart failure risk factors cannot be intervened upon, for example, age and gender. Other risk factors like proinflammatory states may be targets for intervention in the future. This section describes interventions, which directly or indirectly reduce the risk for incident heart failure that is currently known. 

## 6. Lifestyle Changes

Several studies have reported reduced risk for heart failure with healthy lifestyle. Healthy weight, avoiding smoking, engaging in exercise, and a healthy diet have been shown to reduce heart failure risk factors including coronary disease [42, 43], diabetes mellitus [44], and hypertension [45]. Recently, the physicians' health study investigators reported that healthy lifestyle habits, that is, normal body weight, not smoking, regular exercise, moderate alcohol intake, consumption of breakfast cereals, and consumption of fruits and vegetables were associated with a lower risk of heart failure, with the highest risk of 21.3% in men adhering to none of these habits and the lowest risk of 10.1% in men adhering to 4 or more of them [46].

### 6.1. Overweight and Obesity

Body mass index is associated with heart failure in a positive and linear [9] fashion in both sexes. Although body mass index in the obese range (≥30 kg/m^2^) is clearly associated with an increased risk for heart failure [9, 47], there is controversy regarding body mass index in the overweight range (25 to 29.9 kg/m^2^) [47]. Recent data, however, support that overweight is also associated with heart failure [46, 48]. Abdominal obesity may be a stronger predictor for heart failure than total obesity [49], even in the absence of coronary heart disease [50]. Several mechanisms by which elevated body mass index increases the risk of heart failure have been proposed [51, 52] including (a) alterations in cardiac loading, (b) changes in cardiac structure and function [29], (c) activation of neurohumoral and inflammatory pathways [51], (d) promotion of atherogenic conditions [53], (e) predisposition to sleep-disordered breathing [54], and (f) chronic kidney disease [51]. The principal approach of risk reduction in obese patients should include weight control and physical activity, and control of the associated risk factors such as hypertension, diabetes mellitus, sleep disorders, and components of the metabolic syndrome [55]. Myocardial changes with non-surgical or surgical weight loss are feasible and minor weight loss is efficacious; a 10% weight reduction ameliorates systolic dysfunction, and weight loss of 8 to 10 kg produces a significant decrease in left ventricular dimensions and mass index and improves diastolic function. Substantial weight loss reduces left ventricular wall thickness and volume, filling pressures, and improved diastolic measures and improves left ventricular systolic function [55]. The role of metabolic and neurohumoral modification may take precedence over the hemodynamic effects as left ventricular mass or functional improvement occurs independently of loading alterations [56]. 

### 6.2. Sedentary Exercise Habits

Physical inactivity is an important risk factor for cardiovascular diseases including heart failure [48]. Regular physical activity has important and wide-ranging benefits like reduction in risk of cardiovascular diseases [57], hypertension [58], and diabetes [57, 59–61]. Physical activity is a key determinant of good health and an important component of weight reduction and weight maintenance [62], improved lipoprotein profile [62, 63], and reduced risk of hypertension [62], diabetes mellitus [44], and coronary artery disease [62]. These favorable influences on cardiovascular risk profile in turn reduce the likelihood of heart failure. Physical activity could also reduce left ventricular hypertrophy and improve endothelial function [64]. Chronic physical activity reduces cytokine production by adipose tissue, skeletal muscles, and endothelial and blood mononuclear cells and up regulates antioxidant enzymes [65]. These modifying effects on heart failure risk factors or intermediate pathways leading to heart failure can reduce incident heart failure. The integration of physical activity into the daily lives of the population has proved to be challenging. Currently, the recommendations of the American College of Sports Medicine and the American Heart Association for regular physical activity in healthy adults 18–65 years include the following [66].

- *Aerobic Activity. *Moderate-intensity aerobic physical activity for a minimum of 30 min on five days each week or vigorous-intensity aerobic activity for a minimum of 20 min on three days each week.
- *Muscle-Strengthening Activity. *It is recommended that 8–10 exercises should be performed on two or more nonconsecutive days each week using the major muscle groups. To maximize strength development, a resistance (weight) should be used that allows 8–12 repetitions of each exercise resulting in volitional fatigue.
- *Activity Dose. *Vigorous-intensity activities may have greater benefit than moderate-intensity physical activity.

Walking have been reported as beneficial regarding primary prevention [67], this should be adopted by individuals who do not adhere to the current recommendations. 

### 6.3. Alcohol Consumption

Excessive alcohol consumption is associated with alcoholic cardiomyopathy [10, 68]. Interestingly, other data are consistent with possible benefits of moderate alcohol consumption on the risk of heart failure. The New Haven Epidemiologic Study of the Elderly program and the Cardiovascular Health Study reported a 47% [11] and a 34% [69] lower heart failure risk, respectively. The Framingham heart study reported a 59% lower risk among men who consumed 8 to 14 drinks per week compared with abstainers and only a modest and nonsignificant association in women [70]. Moreover, it has been reported that light-to-moderate alcohol consumption is associated with 40% to 50% lower risk of heart failure with previous myocardial infarction, whereas in the same study the risk of heart failure without antecedent myocardial infarction among heavy drinkers was 1.7-fold higher than in abstainers [71]. Similar findings were reported in the physicians' health study [12]. Beneficial effects of alcohol have also been reported on risk for hypertension [72], myocardial infarction, and diabetes mellitus [73], whereas alcohol seems to raise high-density lipoprotein cholesterol [74], improve insulin sensitivity [73], lower plasma levels of inflammatory markers and coagulation factors, and raise plasma levels of adiponectin [10]. 

### 6.4. Dietary Habits

In the Dietary Approaches to Stop Hypertension (DASH) diet, individuals are encouraged to consume more (i) fruits and vegetables, (ii) grains and grain products, (iii) lean meats, fish, poultry, (iv) low fat or nonfat dairy foods, and (v) nuts, seeds, and legumes, and reduce the consumption of red meat, fat, and sugar while maintaining a low sodium intake. Initially, this was promoted for hypertension; however, recent evidence supports reduction of heart failure risk with an observed 37% lower rate in women who adhere to the DASH diet [30]. The DASH diet may contribute to heart failure prevention by reduction in blood pressure [45] and coronary heart disease [45]. Significantly, the DASH diet reduces low-density lipoprotein cholesterol levels and oxidative stress and exerts beneficial physiologic effects like estrogenic effects of phytochemicals [30]. Daily consumption of whole-grain breakfast cereals was associated with a 30% lower rate of heart failure [75], consumption of eggs more than twice per day was associated with a 64% higher rate [76], consumption of fish was associated with a 20–31% lower heart failure rate depending on consumption [77], and consumption of 100 mmol or more of sodium was associated with a 26% higher rate [78]; only nut consumption [79] was not associated with heart failure [80, 81]. When animals were fed the high-fructose diet they demonstrated more cardiac remodeling and worse survival [82]. Whole grain cereals could protect against heart failure risk through effects on weight, hypertension, myocardial infarction, and diabetes mellitus. Nutrients contained in whole grain cereals, for example, potassium, may lower blood pressure, phytoestrogens may improve lipid levels and insulin sensitivity, and other constituents exert beneficial effects on lipid and homocysteine levels [75]. 

Fish consumption exerts beneficial effects on heart failure risk with about a 20% lower risk associated with an intake of 1 to 2 times per week and about a 30% lower risk with intake ≥3 times per week, compared with intake less than 1 time per month. Estimated intake of marine n-3 fatty acids was associated with 37% lower heart failure risk in the highest quintile of intake compared with the lowest. Short-term trials of fish oil supplementation of 3–5 grams per day may reduce risk whereas dietary doses of about 0.5 grams per day may result in more modest effects that over long term may reduce heart failure risk. It has been reported that broiled or baked fish consumption is inversely associated with systolic blood pressure, C-reactive protein levels, and carotid intimal medial thickness, whereas fried fish intake is positively associated with them, indicating that the type of cooking could impact the effects [77].

Historically, human ancestors consumed less than 0.25 grams of salt per day [83]. The recent change to the high salt intake of 10–12 g per day presents a challenge to the physiological systems to excrete these large amounts of salt resulting in a rise in blood pressure and increase in the risk for cardiovascular and renal disease. Currently, the Department of Health and Human Services and Department of Agriculture recommends that adults should consume no more than 2300 mg per day of sodium, but specific groups, that is, all persons with hypertension, all middle-aged and older adults, and all blacks, should consume no more than 1500 mg per day of sodium. Overall, 69.2% of the United States adults meet the criteria for the risk groups. There is overwhelming evidence for a causal relationship between salt intake and blood pressure [84]. A reduction in salt intake may have other beneficial effects on the cardiovascular system, independent of and additive to its effect on blood pressure and include regression of left ventricular hypertrophy, delay in deterioration of renal function, and reduction in proteinuria [85, 86]. 

### 6.5. Smoking

Smoking is a strong predictor of heart failure in both men and women, with 45% and 88% increased risk [87]. The deleterious effect of tobacco seems to be independent of the form of use; increased risk for cardiovascular diseases is reported in nonsmoking use of tobacco [88]. There is no “safe” level of smoking; single cigarette may stiffen the left ventricle [89], and as few as 1 to 4 cigarettes a day double the risk of myocardial infarction [90]. Mechanisms leading to heart failure in smokers include (i) indirect effects, that is by causing or aggravating comorbidities that are related with heart failure, and (ii) direct effects on the myocardium [91, 92]. In animal models, nicotine induces interstitial fibrosis in the ventricles [93]. Besides nicotine, carbon monoxide is also a significant component of tobacco smoke and causes overexpression of growth-related proteins such as calmodulin, calcineurin, and vascular endothelial growth factor [94]. Smoking is associated with higher left ventricular mass, lower stroke volume, ejection fraction [95], and impaired ventricular diastolic function [96]. 

All smokers should be counseled to quit. Patients should be referred to formal cessation programs, and pharmacological therapy should be offered to increase the success rate. Current recommended strategies include the following:

- *Medications*. Several medications are available for tobacco dependence. Seven first-line medications reliably increase long-term smoking abstinence rates including bupropion SR, nicotine gum or inhaler or lozenge or nasal spray or patch, and varenicline.
- *Counseling and Psychosocial Support*. Individual, group, telephone practical counseling, and social support are effective, and their effectiveness increases with treatment intensity.
- *Combination*. The combination of counseling and medication, however, is more effective than either alone, therefore, clinicians should encourage all individuals making a quit attempt to use both counseling and medication.

In the studies of left ventricular dysfunction trials, the risk for heart failure hospitalizations and myocardial infarctions was reduced after quitting [87]. Women's risk of heart disease is reduced by one third within 2 years of quitting and about two thirds within 5 years [97]. 

## 7. Hypertension

Hypertension is an antecedent condition in the majority of individuals developing heart failure [26]. By age 75, almost all hypertensive individuals have isolated systolic hypertension [98]. Diastolic blood pressure is a more potent cardiovascular risk factor than systolic blood pressure till the age of 50, and thereafter systolic blood pressure becomes more important [99]. Clinical trials have shown that controlling systolic hypertension reduces heart failure rates [100]. The population attributable risk of hypertension for heart failure in the general population is reported to be 39% in men and 59% in women by the Framingham investigators [101], whereas population attributable risk of uncontrolled blood pressure in elderly was reported to be 21.3% in whites to 30.1% in blacks in the health, aging, and body composition study [4]. The lifetime risk for heart failure doubles in subjects with blood pressure >160/100 versus those with <140/90 mmHg, and this gradient of risk is seen in both sexes in every decade from 40 to 70 years [102–104]. 

The progression from hypertension to structural ventricular changes and systolic and diastolic ventricular dysfunction is well established. Increases in cardiac afterload, left ventricular mass, and wall stress accompanied by impairment of diastolic filling properties occur in the chronic setting [105–107]. The disproportionately increased left ventricular mass leads to inadequate microvasculature to perfuse the hypertrophied myocardium resulting in subendocardial hypoperfusion, ischemia [108]. These changes increase the risk for coronary thrombosis and infarction characterized by loss of contractile function, neurohormonal activation, and ventricular remodeling leading to the development of systolic dysfunction [109]. Abnormalities in the neurohormonal activation and water and electrolyte balance also play a role in the cascade that leads from hypertension to heart failure [110]. Angiotensin II is an important initiator of matrix remodeling [110, 111], which contributes to the pathogenesis of atherosclerosis and cardiac hypertrophy [110]. The heightened sympathetic nervous system predisposes to vasoconstriction, sodium retention, and ventricular hypertrophy [110]. 

The placebo-controlled trials and the meta-analysis arising from them demonstrate the benefit of antihypertensive therapy in reducing the incidence of cardiovascular diseases [112]. Fewer studies have specifically focused on prevention of left ventricular hypertrophy and development of heart failure. Systolic Hypertension in the Elderly Program (SHEP) demonstrated that antihypertensive treatment when compared to placebo exerted a strong protective effect [100], while a meta-analysis of twelve hypertension trials that included the development of heart failure and four that included the incidence of left ventricular hypertrophy as endpoints demonstrated significant treatment benefits [113]. The incidence of left ventricular hypertrophy was decreased by 35% (95% CI, 21–48%) and the incidence of heart failure was reduced by 52% (95% CI, 41–62%) compared to placebo subjects.

### 7.1. Antihypertensive Medications

- *Diuretics*. Secondary outcomes of the antihypertensive and lipid-lowering treatment to prevent heart attack trial reported a higher rate of incident heart failure with amlodipine (relative risk of 1.35) and a nonsignificantly increase with lisinopril (relative risk of 1.09) compared with chlorthalidone [114]. On the contrary, the second Australian national blood pressure trial [115] reported better outcomes with a regimen that was initiated with an angiotensin converting enzyme inhibitor compared with a diuretic. Diuretics are at least as good as other classes of drugs and also enhance the antihypertensive efficacy of multidrug regimens; Joint National Commission 7 recommend that in the absence of any other compelling indications, thiazide diuretics should be used as initial therapy for hypertension.
- *Renin-Angiotensin System Modulators*. Meta-analysis of double-blind trials that measured the effects of antihypertensive drugs on left ventricular mass [116] shows that the greatest reduction was achieved with angiotensin receptor blockers [117–119]. A recent meta-analysis on renin-angiotensin system inhibition showed that these agents reduce the risk for heart failure by 19% compared with calcium channel blockers [120].
- *Beta-Blockers*. Although beta-blockers are effective in lowering blood pressure, these medications are less effective in preventing hypertension complications including coronary artery disease, cardiovascular, and all-cause mortality [121, 122], or in reducing left ventricular mass [118]. However, a recent meta-analysis suggested that beta-blockers are efficacious for primary prevention of heart failure in hypertension and had a similar benefit in the elderly and younger individuals when compared with other agents [123].
- *Calcium Channel Blockers*. There are limited experimental data on the effects of calcium antagonists on left ventricular mass or incident heart failure. A recent meta-analysis suggested that treatment of hypertension with calcium channel blockers is less effective for reducing heart failure for the same reduction of blood pressure [120].

### 7.2. Target Goals of Therapy

In patients with hypertension, systolic and diastolic blood pressure targets are <140/90 mm Hg except for patients with diabetes or renal disease where the goal is <130/80 mm Hg. Since most patients with hypertension, especially those over age 50, will reach the diastolic blood pressure goal once systolic blood pressure is at goal, the primary focus should therefore be on systolic blood pressure. Recent trials have demonstrated that effective blood pressure control can be achieved in most patients, but the majority will require two or more medications in combination [124]. Data from a recent randomized trial evaluating the effect of usual versus tight control of systolic blood pressure (<130 mm Hg) in nondiabetic hypertensive individuals with left ventricular hypertrophy demonstrated additional benefit with tighter control [125].

## 8. Diabetes Mellitus

Diabetes mellitus is an independent risk factor for heart failure in all age groups [6, 25]. The relative risk for heart failure among patients with diabetes mellitus ranges from 1.3 to 2.7, increasing to 4 in patients younger than 65 years and 11 in those younger than 45 years specifically. Several mechanisms have been proposed to explain the increased risk. Comorbidities associated with heart failure, including obesity, hypertension, and coronary artery disease, are highly prevalent among individuals with diabetes mellitus. Insulin resistance itself may produce abnormalities in cardiac structure and function [126]. Patients with insulin resistance exhibit endothelial dysfunction and a proinflammatory state, which contribute to ventricular dysfunction, even before the development of overt diabetes mellitus. Left ventricular hypertrophy and left ventricular dysfunction are, also strongly correlated with insulin resistance, and hyperinsulinemia has been associated with sympathetic nervous system activation [126]. 

Several mechanisms proposed for the development of “the following diabetic cardiomyopathy” include [127]: (a) microangiopathy and endothelial dysfunction, (b) autonomic neuropathy, (c) metabolic derangements caused by insulin resistance [128], (d) abnormalities in ion homeostasis through alteration of ion channels such as calcium and potassium channels, (e) upregulation of the renin-angiotensin system, (f) increased oxidative stress, (g) increased glycation of interstitial proteins such as collagen creating advanced glycosylation end products, and (h) activation of protein kinase C.

### 8.1. Interventions

#### 8.1.1. Medications

- *Insulin*. Randomized controlled trials indicate that insulin use in ACC-AHA stage. A heart failure does not appear to increase the risk for heart failure [129], whether insulin use specifically reduces the risk for heart failure is not known.
- *Sulfonylureas*. Sulfonylurea therapy does not increase the risk of heart failure compared with other oral antidiabetic agents [130].
- *Metformin*. The risk of new onset heart failure among patients treated with metformin compared with patients treated with other oral antidiabetic medications was reported in ADOPT and the findings were similar as for sulfonylureas [130].
- *Thiazolidinediones*. Although treatment with thiazolidinediones increased myocardial glucose uptake, and myocardial glucose uptake seems to be positively correlated with left ventricular function [128], the rosiglitazone evaluated for cardiac outcomes and regulation of glycaemia in diabetes (RECORD) study indicated that thiazolidinedione use is associated with a small but clinically relevant increased risk of heart failure [20].
- *Other Agents*. There are limited data regarding other antidiabetic therapies for incident heart failure, except for the *α*-glucosidase inhibitor acarbose [131]. Although the study was not powered to evaluate the impact of acarbose treatment on heart failure, the available evidence suggests that this agent may decrease the risk of myocardial infarction [131].

#### 8.1.2. Glucose Control

 In the United Kingdom prospective diabetes study (UKPDS) 33, no significant reduction in the development of macrovascular disease or heart failure was demonstrated with intensive blood glucose control [129].

#### 8.1.3. Blood Pressure Control

 Because hypertension further increases the risk of cardiovascular disease and heart failure in patients with diabetes, aggressive blood pressure management is essential to prevent long-term complications in this population. In UKPDS 38, tight blood pressure control reduced the risk for heart failure by 56% [132]. The seventh report of joint national commission on prevention, detection, evaluation, and treatment of high blood pressure (JNC-7) recommended more aggressive blood pressure control (target blood pressure <130/80 mm Hg) in patients with diabetes [133].

#### 8.1.4. Targeting Underlying Mechanisms

Medications like ACEI, ARB, and beta-blockers benefit patients with diabetes and prevent complications of diabetes including heart failure. The heart outcomes prevention evaluation (HOPE) trial [134], and the microalbuminuria, cardiovascular, and renal outcomes (MICRO-HOPE) [135], have shown that treatment with ACEI reduces the relative risk for new onset heart failure by 23% and 20%, whereas the extension of HOPE study (extended follow-up period) revealed that the benefit of ACEI for heart failure prevention is sustained over time [136]. Likewise, the reduction of endpoints in non-insulin dependent diabetes mellitus with the angiotensin II antagonist losartan (RENAAL) study [137] and the losartan intervention for endpoint reductions in hypertension (LIFE) study [138] have shown a 32% and 41% reduction in the frequency of hospitalization for heart failure, respectively. 

Recently, a randomized trial evaluated the effects of the Cu(II)-selective chelator trientine on left ventricular hypertrophy in diabetes mellitus and showed a 10% decrease in left ventricular mass index after 12 months [139]. This decrease represented a ~50% restoration of left ventricular mass towards normal. In recent studies, breakers of advanced glycation end products related protein cross-links ameliorated the adverse cardiovascular and renal changes associated with aging, diabetes, and hypertension [140]. 

## 9. Coronary Heart Disease

Coronary heart disease predisposes an increased risk for developing heart failure in both men and women [34], with reported population attributable risks ranging from 62% in men to 56% in women [6] and from 23.9% in whites to 29.5% in blacks as reported in the health, aging, and body composition study [4]. Advances in the treatment of myocardial infarction [141] has led to increasing numbers of patients surviving with residual myocardial damage and may be partially responsible for the increase in heart failure incidence among men [6].

### 9.1. Acute Myocardial Infarction

Acute myocardial infarction leads to a cascade of adaptive mechanisms that promote left ventricular remodeling [142]. Ventricular remodeling may continue for weeks or months until the distending forces are counterbalanced by the tensile strength of the collagen scar; this balance then determines the size, location, and transmurality of the infarct, the extent of myocardial stunning, ventricular loading conditions, and local trophic factors [143]. Although contemporary treatment attenuates remodeling [144], there is a large heterogeneity in the remodeling response after an infarction. Reperfusion therapy helps prevent infarct progression; however, it is associated with generation of reactive oxygen species, local inflammatory and oxidant response to reperfusion, and opening of the mitochondrial permeability transition pore that extend infarct size beyond those observed during equivalent periods of ischemia alone [145], Thus, reperfusion injury is a possible target for interventions to reduce myocardial damage. 

### 9.2. Chronic Coronary Artery Disease

Ischemia caused by abnormalities in coronary arteries can produce increases in the concentration of neurohormones, for example, norepinephrine, epinephrine, endothelin, and dopamine, that results in myocardial apoptosis, fibrosis, and susceptibility to ventricular arrhythmias [146]. Thus, ischemia contributes to the progression of left ventricular systolic dysfunction even in the absence of a manifest infarct event. Chronic ischemia can result in hibernation or stunning with further progressive decline in ventricular function. These adaptive-protective mechanisms may result in myocardium rendered hypocontractile and contribute to overall left ventricular systolic dysfunction [146]. Most patients with heart failure from ischemic origin have a substantial volume of myocardium that fails to contract because it is stunned or hibernating rather than because it is scarred. In addition, endothelial dysfunction, an inherent component of the pathophysiology of atherosclerosis, could directly affect ventricular function [147]. Ischemic mitral regurgitation, caused by changes in ventricular structure and function, increases left ventricular preload, leading finally to alteration of left ventricle geometry and deterioration of its function [148]. Moreover myocardial ischemia induces diastolic dysfunction [149] and through alteration of the myocardial passive compliance resulting from scarring, fibrosis, and compensatory hypertrophy of noninfarcted myocardium [150]. 

### 9.3. Interventions

Prevention of coronary heart disease and ischemic events is a key point to maintaining functional myocyte reserve; however, in patients with established coronary heart disease, an aggressive management can reduce development of heart failure. A number of cardioprotective medications and procedures can prevent development of symptomatic heart failure in coronary heart disease. The combination of medications along with therapeutic lifestyle changes should be applied aggressively in all patients to reduce the risk of heart failure.

#### 9.3.1. Revascularization

Mechanical (percutaneous or surgical) or pharmacological revascularization of the infarct-related artery and reduces the size of the acute infarct and prevents subsequent heart failure [144, 151] if performed early enough for myocardial salvage. In addition, the “open artery hypothesis” proposes that late reperfusion, beyond the window for myocardial salvage, also reduces left ventricular remodeling [152]. 

#### 9.3.2. Angiotensin-Converting Enzyme Inhibitors

Angiotensin-converting enzyme inhibitors have favorable properties in reducing left ventricular stress and progression of left ventricle enlargement. In the third Gruppo Italiano per lo Studio della Sopravvivenza nell'Infarto Miocardico (GISSI) trial, early lisinopril therapy in acute infarction reduced mortality and left ventricular dysfunction despite therapy with aspirin, thrombolytics, and beta-adrenergic blocking agents. Similar findings were demonstrated in the survival of myocardial infarction long-term evaluation trial with zofenopril [153]. The Heart outcomes prevention evaluation study demonstrated a 23% reduction in risk for heart failure by ramipril in individuals with established vascular disease [134], expanding the indication for angiotensin-converting enzyme inhibitor therapy to all patients with documented coronary heart disease, presumed coronary heart disease based on presence of other atherosclerotic vascular disease, or diabetes. The European trial on reduction of cardiac events with perindopril in stable coronary artery disease (EUROPA) showed similar data with perindopril [154]. In the survival and ventricular enlargement (SAVE) trial that enrolled patients with asymptomatic left ventricular dysfunction, captopril leads to a 22% reduction in the risk of heart failure hospitalization [155].

#### 9.3.3. Angiotensin-Receptor Blockers

Angiotensin-receptor blockers are at least equally effective as angiotensin-converting enzyme inhibitors in reducing mortality in patients with myocardial infarction complicated by left ventricular dysfunction or heart failure [143, 146]. However, data on populations with atherosclerotic diseases but without heart failure is not uniform [156, 157]. However, since the overall evidence for the effectiveness of angiotensin-receptor blockers on prevention and attenuation of postmyocardial infarction left ventricular remodeling is weaker compared with angiotensin-converting enzyme inhibitors, they may not be used as first-line therapy but will be limited to those individuals who do not tolerate angiotensin-converting enzyme inhibitors.

#### 9.3.4. Beta-Blockers

Beta-blockers have been proven to be beneficial after acute myocardial infarction for over 20 years. Notably, long-term beta-blocker use is recommended for secondary prevention in patients at the highest risk, for example, those with low ejection fraction or heart failure [158]. The reversal of ventricular remodeling with toprol-XL (REVERT) trial gives further evidence that beta-blocker use for the treatment of asymptomatic left ventricular dysfunction to prevent development of heart failure is effective and that left ventricular remodeling can be reversed [159].

#### 9.3.5. Aldosterone Antagonists

Spironolactone combined with angiotensin-converting enzyme inhibitors ameliorated left ventricle remodeling after acute myocardial infarction [143]. Aldosterone antagonists are recommended in myocardial infarction complicated by left ventricular dysfunction, based on the beneficial effect on mortality and cardiovascular hospitalizations seen in the Eplerenone postacute myocardial infarction heart failure efficacy and survival study (EPHESUS) [160].

#### 9.3.6. Antiplatelet Agents

Aspirin in patients with established vascular disease has been demonstrated to reduce risk for cardiovascular events and heart failure [146] and is recommended after acute myocardial infarction and should continue indefinitely if no contraindications exist [161]. 

#### 9.3.7. Statins

Statins are of proven benefit in patients with coronary heart disease [146]; however, their usefulness in the setting of left ventricular dysfunction that remains under investigation. Preprocedural treatment with a statin before percutaneous coronary intervention is associated with lower levels of periprocedural creatine kinase elevation [143]. Ishii et al. [162]. reported that chronic statin therapy before the onset of the acute event is associated with improved perfusion and reduced myocardial necrosis after the intervention. Kjekshus et al. showed a 11% lower risk of new-onset heart failure in patients with stable coronary heart disease treated with statins [163]. Similar trends were also demonstrated in other studies [164], supporting a role for statins in the prevention of heart failure. 

## 10. Conclusions

Considering the worsening epidemiologic trends and increase in the heart failure prevalence in the society, escalating costs, and continued poor quality of life and outcomes for these patients, the importance of heart failure prevention cannot be overemphasized. This will require efforts in all levels of the prevention spectrum ranging from advocacy efforts to research. Population-level efforts at risk factor prevention and adoption of healthy lifestyle habits are essential to promote overall cardiovascular health and reduce heart failure risk specifically. Towards this direction, the American Heart Association has taken a bold step in defining their 2020 goals that include not only achieving further reductions in mortality due to cardiovascular disease and stroke but also improving the health of the population based on a comprehensive specifically designed metric that includes multiple healthy lifestyle parameters. Lastly, whether treatment goals of heart failure risk factors should be individualized based on any given individuals cumulative risk profile needs further study.

## Figures and Tables

**Table 1:** Independent risk factors for incident heart failure.

Reference	Risk factors
Eriksson et al. [26]	Hypertension, smoking, weight, heart size, T-wave abnormality, heart rate variability, peak expiratory flow rate, and psychological stress

Chen et al. [27]	Gender, age, diabetes, pulse pressure, and body mass index

Kannel et al. [28]	Age, blood pressure, LVH, vital capacity, heart rate, CHD, murmurs, diabetes, cardiomegaly, and body mass index

Gottdiener et al. [25]	Age, gender, cerebrovascular disease, diabetes, blood pressure, FEV_1_, creatinine, C reactive protein, ankle arm index, atrial fibrillation, LVH, abnormal ejection fraction, and ECG- ST-T abnormality

He et al. [6]	Gender, education, physical activity, smoking, weight, hypertension, diabetes, valvular disease, and CHD

Wilhelmsen et al. [31]	Age, family history of infarction, diabetes, history of chest pain, smoking, coffee consumption, alcohol abuse, bloods pressure, and body mass index

Bibbins-Domingo et al. [32]	Diabetes, atrial fibrillation, myocardial infarction, creatinine clearance, blood pressure, smoking, body mass index, left bundle branch block, and LVH

Carr et al. [33]	Age, history of myocardial infarction, vascular disease, atrial fibrillation, urinary albumin/creatinine ratio, alcohol abuse, Cornell product, and body mass index

Butler et al. [34]	Age, coronary heart disease, smoking, blood pressure, heart rate, serum creatinine, fasting glucose, albumin level, and left ventricular hypertrophy on electrocardiogram

CHD: coronary heart disease; ECG: electrocardiogram; EPESE: established populations for epidemiologic studies of the elderly; FEV_1_: forced expiratory volume in the 1st second; LIFE: losartan intervention for endpoint reduction in hypertension; LVH: left ventricular hypertrophy; NHANES: National Health and Nutrition Examination Survey; RENAAL: reduction of endpoints in noninsulin dependent diabetes mellitus with the Angiotensin II antagonist Losartan.
